# Pravastatin reduces steroid-induced osteonecrosis of the femoral head in SHRSP rats

**DOI:** 10.3109/17453674.2011.641103

**Published:** 2012-02-08

**Authors:** Yoshihiro Nozaki, Kenji Kumagai, Noriaki Miyata, Masami Niwa

**Affiliations:** ^1^Department of Orthopedic Surgery, Nagasaki University Graduate School of Biomedical Sciences; ^2^Department of Pharmacology, Nagasaki University Graduate School of Biomedical Sciences, Nagasaki, Japan

## Abstract

**Background and purpose:**

Although the definite cause of steroid-induced osteonecrosis of the femoral head (ONFH) is unknown, peripheral circulatory failure, lipid metabolism disturbance, and increased oxidative stress are considered to be possible causes. We investigated whether pravastatin as a statin treatment reduces (1) the incidence of ONFH, (2) the adipocyte area, and (3) bone marrow changes in the femoral head.

**Methods:**

We divided up 81 thirteen-week-old spontaneously hypertensive stroke-prone (SHRSP)/Izm male rats into 4 groups: a control group (group C), a group given pravastatin (group P), a group given steroid (group S), and a group given both pravastatin and steroid (Group PS). The steroid was administered at 15 weeks of age. Pravastatin, as a statin, was administered in the drinking water for 4 weeks. The rats were killed when 17 weeks old. Osteonecrosis was diagnosed based on histopathological examination. Oxidative stress was assessed from immunostaining.

**Results:**

The incidence of histological osteonecrosis was lower in the groups given pravastatin. The percentage of adipocyte area in the bone marrow was lower in the PS group than in the S group. Immunohistochemical staining for oxidative stress showed that staining was less in the PS group than in the S group. Pravastatin had no effect on the blood-derived biochemical findings on lipid metabolism. However, it reduced the incidence of steroid-induced ONFH in these SHRSP rats. We presume that this occurred by reducing oxidative stress and by reducing the percentage of adipocyte area in the femoral heads.

**Interpretation:**

Our data suggest that pravastatin may be effective in reducing steroid-induced ONFH.

Every year, 3,000 of the 128 million inhabitants in Japan develop osteonecrosis of the femoral head (ONFH), and the number of patients—particularly those treated with steroid therapy—has been increasing over the years (Hirota et al. 1997). The proposed pathogenesis of steroid-induced ONFH includes increased oxidative stress, lipid metabolism disturbance, and disturbances of the coagulation-fibrinolysis system due to steroid hormones (Fisher 1978, Cheras 1997, Ichiseki et al. 2004, 2006). Statins are lipid-lowering, 3-hydroxy-3-methylglutaryl coenzyme A (HMG-CoA) reductase inhibitors (Endo 1992). Some statins have been reported to enhance the antioxidant activity and local lipid kinetics by directly acting on adipocytes and blood vessels, thus reducing the severity and frequency of ONFH (Cui et al. 1997, Pritchett 2001, Ichiseki et al. 2004, 2006, Nishida et al. 2008, Yagi et al. 2008 , Kuribayashi et al. 2010).

ONFH observed in spontaneously hypertensive stroke-prone (SHRSP) rats closely resembles human ONFH, not only histologically but also physiologically (Hirano et al. 1989, Wada et al. 2004, Murata et al. 2007, Suzuki et al. 2008). We have reported a 50% incidence of spontaneous osteonecrosis of the femoral head in SHRSP rats at the age of 16–18 weeks, and they develop ONFH more frequently (up to 90%) with steroid administration (Murata et al. 2007, Suzuki et al. 2008). Unlike rabbits, chickens, and other rats, SHRSP rats are an inbred, established model of ONFH.

In this work, we studied the ability of pravastatin to reduce the incidence of steroid -induced osteonecrosis in SHRSP rats. We also examined whether pravastatin could reduce the number of sites of osteonecrosis development in individual rats that developed osteonecrosis. Finally, we determined whether pravastatin has any effects on disorders of lipid metabolism and lipid peroxidation after corticosteroid administration.

## Material and methods

### Animals

All protocols in this study were followed in accordance with the guidelines of the Animal Care and Use Committee of our institution (date of issue: June 17, 2009; registration number: 0906170768).

We used male SHRSP/Izm rats (SHR Cooperative Research Association, Hamamatsu, Japan) at 13 weeks of age. From our previous results of osteonecrosis frequencies, to detect at least a 50% difference between treatment groups (40% in the control group and 90% in the steroid group) with a power of 80% (α = 0.05) (Aoki 2004), we determined that 14 animals would be required in the S and PS groups. Multiple comparisons were not considered when calculating the sample size. Thus, the real statistical power may have been less than 80%. Rats were divided into 4 groups: a control group (group C, 28 rats), a steroid-administered group (group S, 23 rats), a pravastatin-administered group (group P, 14 rats), and a group given pravastatin and steroid (group PS, 15 rats). More rats were allotted to groups C and S to allow us to become familiar with the experimental procedure, but we present the data from all the rats. The rats were housed in standard rat cages at the animal center; they were fed a semipurified diet and were kept in a room at 24 ± 2°C and a humidity of 55 ± 2%. For groups P and PS, pravastatin sodium powder (Mevalotin; Daiichi Sankyo Co. Ltd., Tokyo, Japan) at a dose of 15 mg/day/kg was administered in the drinking water (40–45 mL/day) from 13 weeks of age for 4 weeks. As a steroid hormone, 4 mg methylprednisolone acetate was injected subcutaneously into the back at 15 weeks of age (corresponding to 1,000 mg/60 kg as a human steroid pulse therapy). The rats were killed at 17 weeks of age; they were first anesthetized with a 3-min inhalation of ether, after which they were given an abdominal injection of pentobarbital solution at (50 mg/kg weight).

### Hematological examination

Blood was collected from the heart and both femoral heads were removed. To investigate the hyperlipidemia-improving effects of pravastatin, the serum levels of total cholesterol (T-cho), high-density lipoprotein (HDL), low-density lipoprotein (LDL), and triglycerides (TG) were determined.

### Tissue preparation

The proximal portions of both femurs were fixed in 10% neutral buffered formalin for 24 h, decalcified using EDTA solutions with constant reaction time and temperature, and embedded in paraffin. Subsequently, 4 sections through the teres ligament were stained with hematoxylin and eosin.

### Evaluation of osteonecrosis

**Figure 1.**
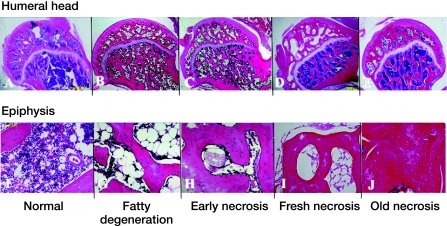
Illustration of histological criteria for ONF. The photomicrographs illustrate normal bone (A, F), fatty degeneration (B, G), early necrosis (C, H), fresh necrosis (D, I), and old necrosis (E, J) (stain: hematoxylin and eosin). The upper row shows whole femoral head (original magnification: ×20) and the lower row shows key findings of each category at high magnification. The fatty degeneration stage has fatty degeneration in the bone marrow (G), the early necrosis stage has myelocyte necrosis in bone marrow (H), the fresh necrosis stage has osteocyte necrosis (I), and the old necrosis stage has appositional bone formation (J).

The histopathological changes were examined with a light microscope. There was no heat generation during section preparation. We evaluated osteonecrosis according to our staging criteria based on Arlet and Durroux (1973), consisting of the presence or absence of fatty degeneration, myelocyte necrosis, osteocyte necrosis, and appositional bone formation (Figure 1).

3 of us (YN, KK, and NM) evaluated all sections by randomly numbering and individually examining all samples to minimize interobserver variability.

### Immunohistochemistry

To detect oxidative stress, specimens were reacted with anti-8-hydroxydeoxyguanosine (anti-8-OHdG) and anti-4-hydroxy-2-nonenal (anti-4-HNE) monoclonal antibodies (NOF Corp, Tokyo, Japan) as primary antibodies for the oxidative stress assay, followed by staining with a Vectastain ABC Kit (Vector Laboratories, Burlingame, CA) according to the manufacturer's instructions. Anti-4-HNE monoclonal antibody detects 4-HNE, which is a secondary product of the oxidation of w-6 polyunsaturated fatty acids, particularly around adipose cells. Use of the anti-8-OHdG monoclonal antibody is based on the fact that when oxidative stress due to active oxygen species is increased, 8-OHdG is formed in DNA and detected in the nucleus. When evaluating the staining for 4-HNE and 8-OHdG, the intensity of staining was also considered. To detect oxidative stress, we examined 20 sections by randomly selecting 5 femoral heads, from separate rats, from each of the 4 groups. Furthermore, to objectively evaluate the findings, we evaluated the staining of cells and surrounding structures in these sections: in the blood vessels, adipocytes, bone marrow cells, and trabeculae in each group using a 3-point scale (negative, positive, and intensely positive) and calculating the mean total scores by a “3-point method”. Also, the images were examined blind, and the scores were averaged among the observers. 3 of the authors (YN, KK, and NM) examined all the immunochemical sections and scored them using the 3-point method.

### Measurement of the adipose area in bone marrow

To measure the percentage of adipose area in the bone marrow, we used a camera connected to an image processor, according to the procedure of Nishida et al. (2008). The area of the bone marrow and adipose cells displayed on the video monitors was measured using an interactive mouse pad-tracing instrument (NIH Image software program; NIH, Bethesda, MD).

### Statistics

All statistical analyses were performed using SAS software version 9.1.3 (SAS Institute Inc., Cary, NC). Data were expressed as mean (SD). To detect differences in the incidence of ONFH between the groups, we used generalized estimating equations (GEEs), a method that allows inclusion of correlated observations, in this case between the right and left heads (Park et al. 2010). For comparisons of means among multiple groups, a general linear modeling method (ANOVA) and Tukey's method for multiple comparisons were used for continuous variables.

## Results

### Osteonecrosis

We analyzed 160 femoral heads in 80 rats (Table 1). The incidence of histological ONFH was higher in group S than in group C (p < 0.001), and lower in group PS than in group S (p < 0.001), indicating that pravastatin markedly inhibits ONFH. The inter-observer variability was about 10%.

**Table:** Incidence of histological ONFH with pravastatin. Mean (SD)

Pravastatin Steroid Group	–	–	+	+
	–	+	–	+
	C	S	P	PS
	(n = 28)	(n = 23)	(n = 14)	(n = 15)
Necrosis, n (%)
0	13 (46)	0 (0)	10 (71)	4 (27)
1	13 (46)	4 (17)	3 (21)	9 (60)
2	2 (7)	19 (83)	1 (7)	2 (13)
p-value		< 0.001 **^a^**	0.3 **^a^**	< 0.001 **^b^** 0.05 **^c^**

**^a^** compared with group C.

**^b^** compared with group S.

**^c^** compared with group P.

### Adipose area in bone marrow

**Figure 2.**
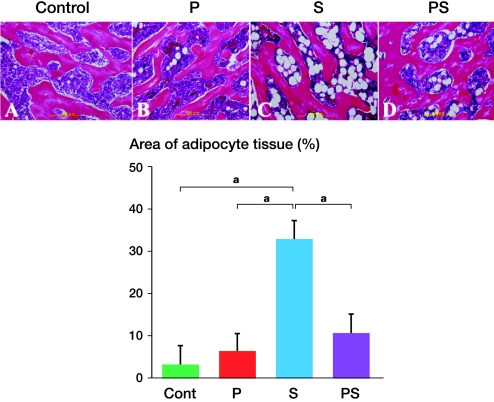
Area of adipocytes in the bone marrow. The pictures in the upper row show that the adipocyte area in the bone marrow of the femoral head epiphysis was larger in group S (panel C) than in groups PS (panel D), C (panel A), or P (panel B) (stain: hematoxylin and eosin; original magnification: ×100). In the lower panel, the percentage area of adipocyte tissue in the bone marrow is compared for the 4 groups (see Results). Error bars indicate SD. **^a^** p < 0.001 (Fisher's exact test).

5 femoral heads, which were not from the same rat, were randomly selected from each of the 4 groups. Of these, the percentage of adipocyte area was higher in group S (33% (SD 9)) than in groups C (3% (SD 3)), P (6% (SD 7)), and PS (11% (SD 5)) (Figure 2). Thus, the adipocyte area was reduced by about 20% as a result of pravastatin treatment. The fact that the percentage in group PS was statistically significantly smaller than that in group S appears to indicate that pravastatin reduced the effect of steroid.

### Oxidative stress

**Figure 3.**
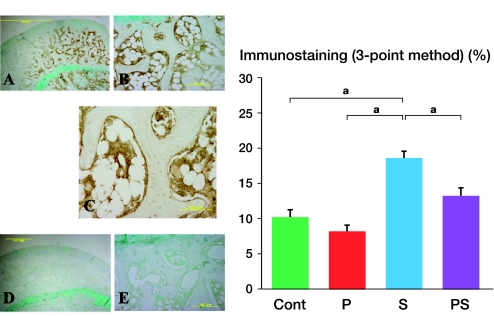
Comparison of oxidative stress (4-HNE). Very strong immunostaining was observed in group S (A, B), but the staining was very weak in group PS (D, E) in comparison. Magnification: ×20 (A, D), ×100 (B, E). C. Strong immunostaining in marrow cells, around adipocytes, and in vessel walls in group S. Magnification: ×200. F. The strongest staining was observed in group S, followed by group PS, group C, and group P with the 3-point method. Error bars indicate SD. **^a^** p < 0.001 (Fisher's exact test).

When oxidative stress was compared using staining with anti-4-HNE and anti-8-OHdG monoclonal antibodies, similar tendencies with weaker changes were observed with anti-8-OHdG. In group S, the adipose cell and myelocytes were strongly stained and some osteocytes were also stained. The staining in the adipose cells was not diffuse, but was localized around the membrane. In the PS group, staining of the adipose cell membrane was weaker than that in the S group. In the C and P groups, little or no staining for oxidative stress in the adipose cell membrane was observed. These findings were more obvious from the results of the 3-point method (Figure 3).

Thus, it appears that bone marrow changes can be suppressed (by about 5%) by pravastatin treatment. The scoring by the 3 observers was consistent.

### Vascular damage

The vascular damage was assessed from hematoxylin and eosin-based histological data from the 4 groups. No statistically significant changes were observed between groups.

### Body weight, laboratory data, and blood pressure.

There was no statistically significant difference in blood pressure between groups C and S; the mean values were the same (284 Torr in both groups). Pravastatin administration caused weight loss (mean 314 g in group C vs. 281 g in group P), but the rats grew normally. None of the rats died due to the steroid administration. Steroid loading resulted in substantial weight loss (mean 314 g in group C vs. 246 g in group S). Blood chemistry data showed that steroid hormone administration (group S) induced marked hyperlipidemia, that is, significantly elevated total cholesterol levels (mean 60 mg/dL in group C vs. 104 mg/dL in group S), HDL levels (mean 18 mg/dL in group C vs. 30 mg/dL in group S), LDL levels (mean 8.3 mg/dL in group C vs. 16 mg/dL in group S), and triglyceride levels (mean 40 mg/dL in group C vs. 102 mg/dL in group S) relative to the C group (p < 0.05 in all cases). Administration of pravastatin in combination with the steroid hormone did not reduce hyperlipidemia or total cholesterol.

## Discussion

Circulatory disturbance, impairment of lipid metabolism, and increased oxidative stress have been suggested to be involved in the etiology of steroid-induced ONFH. Many drugs to inhibit these have been tested in animals and humans, resulting in reports of prevention or suppression of the progression of ONFH by the administration of warfarin, low-molecular-weight heparin, statins, and vitamin E (Pritchett 2001, Glueck et al. 2005, Mont et al. 2006, Nagasawa et al. 2006, Kuribayashi et al. 2010). Glueck et al. (2005) studied 224 patients: 26 patients with multifocal osteonecrosis, 91 patients with unifocal osteonecrosis, and 117 control patients. They found that osteonecrosis was associated with familial thrombophilia and eNOS T-786C polymorphism.

Statins (HMG-CoA reductase inhibitors) have been widely used to treat hyperlipidemia (Endo 1992). In a clinical study of 284 patients with statin, Pritchett (2001) observed improvement in hyperlipidemia and lower incidence of osteonecrosis in patients who were on high-dose steroid treatment, compared with the control group. That study was retrospective, so whether the high lipid level was a cause of—or merely associated with—the osteonecrosis is unclear. Furthermore, Jasiñska et al. (2007) reported pleiotropic (multiphasic) effects of statins such as increased upregulation of eNOS and nitric oxide production. We selected a hydrophilic statin as a prophylactic agent for ONFH in the present study using SHRSP rats.

Our study has some limitations. First, there have been few studies using rats. However, the rats we used were of a pure strain that we have used in various studies, with consistent results. This also contributed to simplification of the study design. Various animal species have been used as experimental models of steroid-induced ONFH, including rabbits, dogs, birds including the emu, and sheep (Li et al. 2009). The SHR rats were developed in 1959 by Okamoto et al., and not only have hypertension but also peripheral circulatory failure (Okamoto 1969, Yamori 1984).

We have conducted research using SHRSP for over 20 years, and have found that the pathology of ONFH in this model resembles that in humans more closely, being localized in the femoral head, than in the more commonly used rabbits (Murata et al. 2007, Suzuki et al. 2008). The second limitation is the extrapolation from rats to humans, and the third is that our study involved a single observation point. As SHRSP rats are very sensitive to stimulation, multiple blood drawing is dangerous for the animals. Also, we considered that a serological comparison between the groups at a single observation point was possible because they are a pure strain of rats that has uniform characteristics. Finally, we used a water-soluble statin, so that the doses of pravastatin could be easily adjusted by dissolving it in drinking water. Multiple feedings or injections are dangerous for SHRSP rats because of their sensitivity. The stronger statins, such as lovastatin, were not suitable for use with SHRSP rats because they have been reported to be insoluble in water (Cui et al. 1997, Li et al. 2003).

Our findings provide further evidence that a statin can suppress steroid-induced ONFH in rats. Nishida et al. (2008) reported that a statin reduced the incidence of steroid-induced ONFH by one third. However, Fujioka et al. (1997) found that cholesterol synthesis in the rat liver increased after 7 days of consecutive administration of pravastatin, and suggested that induced HMG-CoA reductase activity overcomes the inhibitory effects of pravastatin, resulting in an increase in net cholesterol synthesis in rats, but not in rabbits. According to our histological results, the area of bone marrow occupied by adipocytes was reduced by about one fifth, which we interpret as a sign of improvement in local lipid metabolism by pravastatin treatment. Kuribayashi et al. (2010) also found that there is a decrease in local lipid levels after vitamin E administration, even without any reduction in lipid levels in blood tests.

Nishida et al. (2008) noted that not only LDL-lowering effects but also non-lipid effects of pitavastatin contributed to the prevention of ONFH by reducing the formation of thrombi and lipid emboli in bone marrow blood vessels. In a study of the action of statins on adipocytes in bone marrow, Li et al. (2003) reported that lovastatin inhibits adipocyte differentiation, apparently by acting on the expression of the fat cell-specific genes PPARγ2 and 422aP2 and subsequent maturation. Cui et al. (1997) suggested that lovastatin prevents the effects of steroids on adipogenesis and the expression of fat-specific gene 422 (aP2), and attenuates the inhibitory effects of steroids on osteoblastic gene expression in cultured cells and adipogenesis and osteonecrosis in chickens. Yagi et al. (2008) reported that pitavastatin may reduce the oxidative stress induced by reduced nitric oxide bioavailability, and thereby prevent vascular endothelial injury caused by excess corticosteroids—thus being an effective pleiotropic inhibitor of the development of ONFH.An increase in oxidative stress due to steroid administration can be detected immunohistologically, but changes were demonstrated numerically in this study. The reduction in oxidative stress and incidence of ONFH by the statin may have occurred by chance in the present study, but many researchers have recently detected oxidative stress at the sites of ONFH or have demonstrated suppression of osteonecrosis by an antioxidant agent. Ichiseki et al. (2004, 2006) reported that ONFH could be suppressed by controlling oxidative stress in rabbits and rats, and Kuribayashi et al. (2010) described the prevention of ONFH in rabbits by the antioxidant action of vitamin E at a dose that has no effect on lipid metabolism. Some reports have mentioned the oxidative stress-induced endothelial dysfunction and ischemic change in SHR vessels (Nakamura et al. 2007, Yao et al. 2007). It is still not clear which has a greater effect on inducing ONFH—oxidative stress in adipocytes or in vessels—so further investigation will be necessary.

Our preliminary data from this rat model suggest that pravastatin, a water-soluble HMG-CoA reductase inhibitor, may be useful in reducing steroid-induced ONFH.

## References

[CIT0001] Aoki S (2004). http://aoki2.si.gunma-u.ac.jp/lecture/SampleSize/ptest.html.

[CIT0002] Arlet J, Durroux R, Arlet J, Ficat P (1973). Lagnostic histologique precoce de l'osteonecrose aseptique do la tete femorale par le forage biopsie. La Ciculation Osseuse.

[CIT0003] Cheras PA, Urbaniak JR, Jones JP (1997). Role of hyperlipidemia, hypercoagulability, and hypofibrinolysis in osteonecrosis and osteoarthritis. Osteonecrosis: Etiology, Diagnosis, and Treatment.

[CIT0004] Cui Q, Wang GJ, Su CC, Balian G (1997). Lovastatin prevents steroid induced adipogenesis and osteonecrosis. Clin Orthop.

[CIT0005] Endo A (1992). The discovery and development of HMG-CoA reductase inhibitors. J Lipid Res.

[CIT0006] Fisher DE (1978). The role of fat embolism in the etiology of corticosteroid-induced avascular necrosis: clinical and experimental results. Clin Orthop.

[CIT0007] Fujioka T, Tsujita Y, Shimotsu H (1997). Induction of fatty acid synthesis by pravastatin sodium in rat liver and primary hepatocytes. Eur J Pharmacol.

[CIT0008] Glueck CJ, Freiberg RA, Sieve L, Wang P, Enoxaparin prevents progression of Stages I (2005). II osteonecrosis of the hip. Clin Orthop.

[CIT0009] Hirano T, Iwasaki K, Sagara K, Nishimura Y, Kumashiro T (1989). Necrosis of the femoral head in growing rats: occlusion of lateral epiphyseal vessels. Acta Orthop Scand.

[CIT0010] Hirota Y, Hotokebuchi T, Sugioka Y, Urbaniak JR, Jones JP (1997). Idiopathic osteonecrosis of the femoral head: nationwide epidemiologic studies in Japan. Osteonecrosis: Etiology, Diagnosis, and Treatment.

[CIT0011] Ichiseki T, Matsumoto T, Nishino M, Kaneuji A, Katsuda S (2004). Oxidative stress and vascular permeability in steroid-induced osteonecrosis model. J Orthop Sci.

[CIT0012] Ichiseki T, Ueda Y, Katsuda S, Kitamura K, Kaneuji A, Matsumoto T (2006). Oxidative stress by glutathione depletion induces osteonecrosis in rats. Rheumatology.

[CIT0013] Jasinska M, Owczarek J, Orszulak-Michalak D (2007). Statins: a new insight into their mechanisms of action and consequent pleiotropic effects. Pharmacol Rep.

[CIT0014] Kuribayashi M, Fujioka M, Takahashi KA, Arai Y, Ishida M, Goto T, Kubo T (2010). Vitamin E prevents steroid-induced osteonecrosis in rabbits. Acta Orthop.

[CIT0015] Li X, Cui Q, Kao C, Wang GJ, Balian G (2003). Lovastatin inhibits adipogenic and stimulates osteogenic differentiation by suppressing PPARγ2 and increasing Cbfa1/Runx2 expression in bone marrow mesenchymal cell cultures. Bone.

[CIT0016] Li Y, Han R, Geng C, Wang Y, Wei L (2009). A new osteonecrosis animal model of the femoral head induced by microwave heating and repaired with tissue engineered bone. Int Orthop.

[CIT0017] Mont AM, Jones CL, Hungerford SD (2006). Nontraumatic osteonecrosis of the femoral head: ten years later. J Bone Joint Surg (Am).

[CIT0018] Moon Seok Park, Sung Ju Kim, Chin Youb Chung, In Ho Choi, Sang Hyeong Lee, Kyoung Min Lee (2010). Statistical consideration for bilateral cases in orthopaedic research. J Bone Joint Surg (Am).

[CIT0019] Murata M, Kumagai K, Miyata N, Osaki M, Shindo H (2007). Osteonecrosis in stroke-prone spontaneously hypertensive rats: effect of glucocorticoid. J Orthop Sci.

[CIT0020] Nagasawa K, Tada Y, Koarada S, Tsukamoto H, Horiuchi T, Yoshizawa S, Murai K, Ueda A, Haruta Y, Ohta A (2006). Prevention of steroid-induced osteonecrosis of femoral head in systemic lupus erythematosus by anti-coagulant. Lupus.

[CIT0021] Nakamura T, Yamamoto E, Kataoka K, Yamashita T, Tokutomi Y, Dong Y-F, Matsuba S, Ogawa H, Kim-Mitsuyama S (2007). Pioglitazone exerts protective effects against stroke in stroke-prone spontaneously hypertensive rats, Independently of blood pressure. Stroke.

[CIT0022] Nishida K, Yamamoto T, Motomura G, Jingushi S, Iwamoto Y (2008). Pitavastatin may reduce risk of steroid-induced osteonecrosis in rabbits: a preliminary histological study. Clin Orthop.

[CIT0023] Okamoto K (1969). Spontaneous hypertension in rats. Int Rev Exp Pathol.

[CIT0024] Pritchett JW (2001). Statin therapy decreases the risk of osteonecrosis in patients receiving steroids. Clin Orthop.

[CIT0025] Suzuki M, Kumagai K, Osaki M, Murata M, Tomita M, Miyata N, Hozumi A, Niwa M (2008). Osteonecrosis of femoral head in the stroke-prone spontaneously hypertensive rats, especially old rats. Clin Exp Hypertens.

[CIT0026] Wada M, Kumagai K, Murata M, S-Yamashita Y, Shindo H (2004). Warfarin reduces the incidence of osteonecrosis of the femoral head in spontaneously hypertensive rats. J Orthop Sci.

[CIT0027] Yagi S, Aihara K, Ikeda Y, Sumitomo Y, Yoshida S, Ise T, Iwase T, Ishikawa K, Azuma H, Akaike M, Matsumoto T (2008). Pitavastatin, an HMG-CoA reductase inhibitor, exerts eNOS-independent protective actions against angiotensin II induced cardiovascular remodeling and renal insufficiency. Circ Res.

[CIT0028] Yamori Y (1984). Development of the spontaneously hypertensive rat (SHR) and of various spontaneous rat models, and their implications. Handbook of Hypertension.

[CIT0029] Yao EH, Fukuda N, Matsumoto T, Kobayashi N, Katakawa M, Yamamoto C, Tsunemi A, Suzuki R, Ueno T, Matsumoto K (2007). Losartan improves the impaired function of endothelial progenitor cells in hypertension via an antioxidant effect. Hypertens Res.

